# Quantifying absolute glutamate concentrations in nucleus accumbens of prescription opioid addicts by using ^1^H MRS

**DOI:** 10.1002/brb3.769

**Published:** 2017-07-14

**Authors:** Xi‐Long Liu, Long Li, Jian‐Neng Li, Ji‐Hua Tang, Jia‐Hui Rong, Bo Liu, Ze‐Xuan Hu

**Affiliations:** ^1^ Department of Radiology Guangdong Provincial Corps Hospital of Chinese People's Armed Police Forces Guangzhou Medical University Guangzhou China; ^2^ Department of Psychology and Addiction Medicine Guangdong Provincial Corps Hospital of Chinese People's Armed Police Forces Guangzhou Medical University Guangzhou China

**Keywords:** glutamate, impulsivity, magnetic resonance spectroscopy, neural circuits, neurotransmitters, substance use disorders

## Abstract

**Introduction:**

The diagnosis of psychoactive substance use disorders has been based primarily on descriptive, symptomatic checklist criteria. In opioid addiction, there are no objective biological indicators specific enough to guide diagnosis, monitor disease status, and evaluate efficacy of therapeutic interventions. Proton magnetic resonance spectroscopy (^1^H MRS) of the brain has potential to identify and quantify biomarkers for the diagnosis of opioid dependence. The purpose of this study was to detect the absolute glutamate concentration in the nucleus accumbens (NAc) of patients with prescription opioid dependence using ^1^H MRS, and to analyze its clinical associations.

**Methods:**

Twenty patients with clinically diagnosed definitive prescription opioid dependent (mean age = 26.5 ± 4.3 years) and 20 matched healthy controls (mean age = 26.1 ± 3.8 years) participated in this study. Patients were evaluated with the Barratt Impulsiveness Scale (BIS‐11), the Self‐Rating Anxiety Scale (SAS), and the opiate Addiction Severity Inventory (ASI). We used point‐resolved spectroscopy to quantify the absolute concentrations of metabolites (glutamate, choline, *N*‐acetylaspartate, glutamine, creatine) within the NAc. The difference between metabolite levels of groups and Pearson's correlation between glutamate levels and psychometric scores in patients were analyzed statistically.

**Results:**

Glutamate concentrations in the NAc were significantly higher in prescription opiate addicts than in controls (*t *=* *3.84, *p *=* *.001). None of the other metabolites differed significantly between the two groups (all *p*s > .05). The glutamate concentrations correlated positively with BIS‐11 scores in prescription opiate addicts (*r *=* *.671, *p *=* *.001), but not with SAS score and ASI index.

**Conclusions:**

Glutamate levels in the NAc measured quantitatively with in vivo ^1^H MRS could be used as a biomarker to evaluate disease condition in opioid‐dependent patients.

## 1 INTRODUCTION

The misuse of opiates has been one of the most serious societal, economic, and health problems in the world. Opioid dependence, which is characterized by extremely unpleasant physical and emotional feelings after drug use is terminated (Zhu, Wienecke, Nachtrab, & Chen, 2016), is a highly prevalent addictive disorder associated with comorbid medical and psychiatric problems (Rodriguez‐Cintas et al., 2016). Most previous studies of drug addiction's effect on brain systems have focused on illicit drugs, such as heroin, cocaine, cannabis (Greenwald, Woodcock, Khatib, & Stanley, 2015; Paydary et al., 2016; Rodriguez‐Cintas et al., 2016), as opposed to legal opiates (e.g., codeine, morphine, and opium) (Kim, Ham, Hong, Moon, & Im, 2016; Qiu, Su, Lv, & Jiang, 2015; Schuckit, 2016). Long‐term use of prescription opioid exposes patients to risk of developing addictive side effects, such as rewarding and withdrawal symptoms (Kim et al., 2016).

The diagnosis of psychoactive substance use disorder is currently based on subjective judgments of symptomatic checklist criteria. There are no biomarkers of addiction severity and no reliable set of criteria that are specific enough to help guide diagnosis, monitor disease state, and evaluate therapeutic effects (Volkow, Koob, & Baler, 2015).

Neuroimaging in substance use disorders examines neural circuits with regard to both molecular mechanisms and behavior (Garrison & Potenza, 2014). Recent technological advances in neuroimaging have the potential to impact significantly the identification of biomarkers of opioid addiction and its treatment. Proton magnetic resonance spectroscopy (^1^H MRS) is a noninvasive evaluation that provides in vivo quantification of the concentrations of selected biochemicals.

The brain's reward circuitry is the neuroanatomical basis of producing the reward effect in psychoactive substance dependence (Kim et al., 2016; Koob & Volkow, 2010; Russo & Nestler, 2013). The nucleus accumbens (NAc) is one of the most important nuclei in the reward circuitry, and acts as an interface for the transfer of information between the limbic and motor systems (Mavridis, Boviatsis, & Anagnostopoulou, 2011; Zhu et al., 2016). Glutamate is a major excitatory neurotransmitter in reward circuitry. It could be involved in the formation and mediating the long‐term effects of opioid dependence.

This study aimed to detect concentrations of glutamate in the NAc of prescription opioid addicts using ^1^H MRS, and to explore the correlations with clinical indices. We seek an objective scientific basis for the biochemical diagnosis of opioid addiction, and also a basis for assessing the efficacy of intervention strategies.

## 2 MATERIALS AND METHODS

### 2.1 Participant characterization

All participants provided informed consent according to the procedures approved by the Institutional Review Board. Twenty patients who fulfilled the Diagnostic and Statistical Manual of Mental Disorders, 4th Edition (DSM‐IV) criteria for prescription opiate dependence (codeine‐containing cough syrups), along with a urine test and an interview conducted by clinical psychologists, were admitted for inpatient management to the Department of Psychology at Guangdong Provincial Corps Hospital of Chinese People's Armed Police Forces. Twenty healthy control participants were recruited using advertisements in local newspapers. All patients were naïve to use other types of psychoactive drugs and were treatment naive. All study subjects were right handed, and none of all subjects meet the DSM‐IV criteria for alcohol dependent. Patients periodically used cigarettes and denied using psychotropic drugs and alcohol in the month before the MR examination. Exclusion criteria for all participants included psychiatric disorders, neurological disorders, history of serious head injury, and the abuse or dependence on any other substance other than nicotine. The opiate Addiction Severity Inventory (ASI; Luo, Guo, Han, & Li, 2012; Sun et al., 2012) was implemented in the patient group to assess the clinical characteristics of the subjects’ opiate dependence. The Barratt Impulsiveness Scale (BIS‐11; Huang, Li, Fang, Wu, & Liao, 2013; Patton, Stanford, & Barratt, 1995) was implemented as a self‐administered questionnaire to assess subjects’ impulsiveness, and the Self‐Rating Anxiety Scale (SAS) (Olatunji, Deacon, Abramowitz, & Tolin, 2006) was used to measure anxiety symptoms. All the rating scales were surveyed soon after MR imaging. Demographic and clinical characteristics of the patients and controls are summarized in Table 1.

**Table 1:** Demographic and clinical data of the study and control groups

	Patients (*n* = 20) (mean ± SD)	Controls (*n* = 20) (mean ± SD)	Statistics	*p*
Age (years)	26.5 ± 4.3	26.1 ± 3.8	*t* = 0.291	.529
Gender (*N*)				
Male	18	13	χ^2^ = 3.584	.127
Female	2	7		
Education (*N*)				
Junior high school	5	2	χ^2^ = 2.887	.236
Senior high school	8	6		
College/university	7	12		
Duration of opiates dependence (years)	5.3 ± 3.1	N/A	—	—
Mean dose (ml/day)	389.28 (range: 60–1500)	N/A	—	—
Total BIS‐11 score	73.9 ± 3.3	N/A	—	—
Total SAS score	39.5 ± 6.1	N/A	—	—
Total ASI score	16.9 ± 4.4	N/A	—	—
Nicotine (yes:no)	19:1	14:6	χ^2^ = 4.329	.091
Cigarettes/day (smokers only)	15.3 ± 7.5	13.8 ± 4.3	*t* = 0.685	.499

N/A, not applicable; BIS‐11, Barratt Impulsiveness Scale; SAS, Self‐Rating Anxiety Scale; ASI, Addiction Severity Inventory.

### 2.2 ^1^H MRS

**Figure 1:**
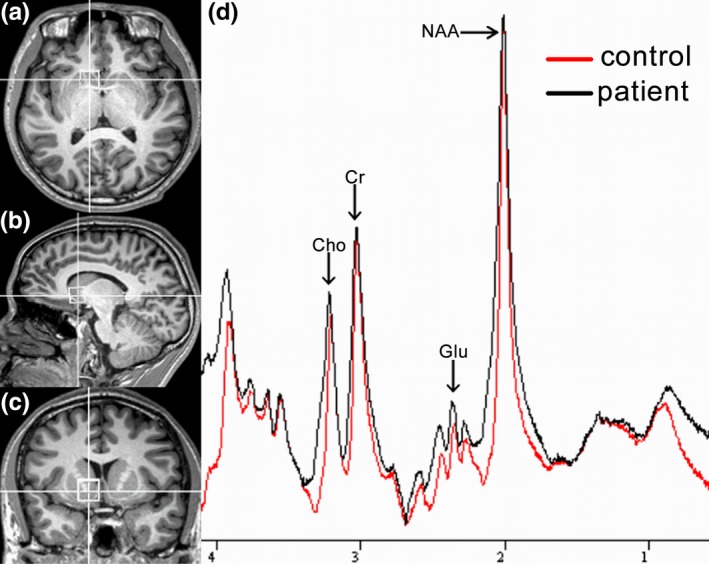
The location and MR spectra of the region of interest in the nucleus accumbens. (a) Localized images of the nucleus accumbens in the axial plane, (b) in the coronal plane, and (c) in the sagittal plane; (d) results of MR spectra of nucleus accumbens in the patient (black line) and control (red line) brain

All subjects were scanned in a MAGNETOM Skyra 3T MRI Scanner (Siemens Healthineers, Erlangen, Germany) with a 20‐channel phased‐array joint head and neck coil. Foam padding and a forehead‐restraining strap were utilized to limit the head movement of subjects during the scanning procedure. To obtain high‐quality spectroscopy data, participants were advised of the importance of remaining motionless during the procedure. T_1_‐weighted high‐resolution anatomical images of the whole brain were acquired using a three‐dimensional fast gradient echo sequence: TR = 2,300 ms, TE = 2.98 ms, TI = 900 ms, FOV = 256 × 256 mm, slice thickness = 1.1 mm, flip angle = 9°, TA = 5 min 12 s. These slices in three orthogonal planes were displayed using multiplanar reconstruction for localization of the spectroscopic volumes of interest (VOI: 15 × 15 × 15 mm = 3.4 cm^3^). The NAc is the main part of the ventral striatum. Thus, the VOI was positioned to cover the most ventral part of the striatum in the coronal and sagittal slices with the ventral corner of the lateral ventricle as a topographic marker point (Figure 1).

All MRS data showed were acquired using single‐volume localization. Spectral data were acquired with conventional PRESS spectra using a TE of 40 ms. A short echo time was chosen to obtain the optimal selectivity for glutamate (Hancu, 2009; Mullins, Chen, Xu, Caprihan, & Gasparovic, 2008; Wijtenburg & Knight‐Scott, 2011). The spectral pattern for glutamate and glutamine at a TE of 40 ms has been depicted by Mullins et al. (2008). These spectra were also collected with a TR of 2,000 ms, with 128 averages, giving a total scan time of over 4.5 min. The raw data from each acquisition consisted of 1,024 points at a bandwidth of 1,200 Hz. The total examination time was approximately 10 min. For quality control, a phantom with a concentration of 50 mmol/L creatine was measured using the same protocol during each MRS session.

### 2.3 MRS data analysis

Quantification of the spectra was based on jMRUI software (http://www.mrui.uab.es/mrui/). jMRUI enables time domain analysis of in vivo MRS data, which subdivided into two stages: preprocessing and quantitation (Naressi, Couturier, Castang, de Beer, & Graveron‐Demilly, 2001; Stefan et al., 2009). In preprocessing, it is supported user interaction. The procedure included zero filling to 2,048 points, slight apodization, varying from 2 to 4 Hz. HLAVD filters are largely used to suppress residual water molecules and the cadzow function used to filter the signal. Because the preprocessing step is manual, the results of model fitting may be influenced and thus affected the accuracy of the signal quantification. This procedure was done by a neuroradiologist. The metabolite peaks of interest were quantified using the advanced method for accurate, robust, and efficient spectral fitting (AMARES) algorithm. To improve the quantification process, this method requires the user to input of prior knowledge of the estimated peaks. In this study, all analyses peaks (*N*‐acetylaspartate, glutamate, glutamine, creatine, choline) positions were set 2.02 parts per million (ppm) and 3.9 line width (LW [Hz]), 2.35 ppm and 4.9 LW, 2.45 ppm and 4.9 LW, 3.01 ppm and 4.9 LW, 3.20 ppm and 4.9 LW, respectively (Scott, Underwood, Garvey, Mora‐Peris, & Winston, 2016). The AMARES method provided estimates for the peak frequency, amplitude, phase, and line width of the metabolism resonances. The concentrations were calculated according to previously reported and as described in detail by Helms (2008). In order to take into consideration of the temperature and relaxation times (T_1_ and T_2_) effects of metabolites of interest in vivo and in vitro, calibrations were performed. For simplicity, we used the reported values from the literature (Choi et al., 2006; Mlynarik, Gruber, & Moser, 2001; Traber, Block, Lamerichs, Gieseke, & Schild, 2004). Because the T_1_ and T_2_ value in NAc has not yet been reported, we used T_1_ and T_2_ values calculated as an average of the literature reported values in basal ganglia. Briefly, the T_1_ values of *N*‐acetylaspartate, creatine, choline, glutamate, and glutamine were 1.39, 1.47, 1.15, 1.22, and 1.22 s, and the T_2_ values were 221, 143, 201, 199, and 199 ms, respectively. Cramer–Rao lower bounds (CRLBs) were invoked as a measure of the accuracy of the calculation of the amplitude of a certain component. CRLBs were estimates of the %*SD* of the fit for each metabolite. Only metabolite concentrations with CRLBs below 20% were accepted and used for the following analyses.

### 2.4 Statistics

Statistical calculations were carried out using SPSS 13.0. The metabolite levels and the clinical and demographical variables were analyzed with two‐sided *t*‐test for independent samples and chi‐square test, respectively. The data distributions were tested for normality using P‐P plots and the Kolmogorov–Smirnov test. Correlations of metabolite levels and clinical characteristics were evaluated as Pearson's coefficients. All tests were classified as significant if the *p* < .05.

## 3 RESULTS

### 3.1 Demographic and clinical characteristics

The demographic and clinical data are summarized in Table 1. Overall, there were no significant differences between the two groups (all *p* > .05; Table 1). The psychometric scores include BIS‐11, ASI, and SAS were measured in the prescription opiate‐dependent group. The possibly confounding variable of nicotine consumption (cigarettes per day) was balanced between the groups.

### 3.2 MRS results

**Figure 2:**
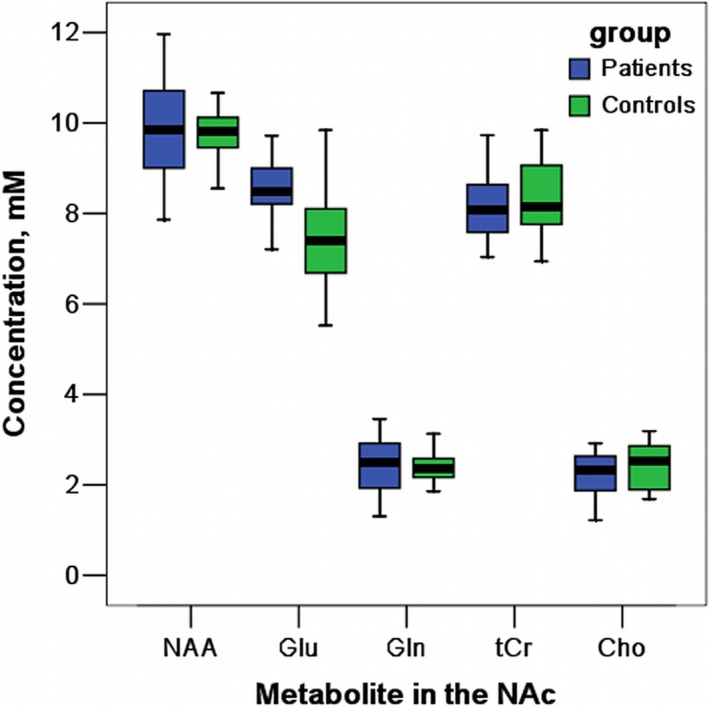
Boxplot for major metabolite concentrations in the nucleus accumbens (NAc)

The CRLBs of *N*‐acetylaspartate, glutamate, creatine, and choline were less than 20% on all participants, whereas the glutamine data from 1 control subject and 2 prescription opiate users were excluded due to excessive CRLBs. The mean metabolite concentrations in the NAc are displayed in Table 2 and Figure 2. We found significantly higher glutamate concentrations in the NAc in the prescription opiate‐dependent patients relative to the controls, *t*(38) = 3.84, *p* = .001. None of the other metabolites differed significantly between the two groups (all *p* > .05; Table 2).

**Table 2:** Comparison of absolute metabolite concentrations in the nucleus accumbens (mmol/L) between the prescription opioid‐dependent and healthy control group

Metabolite	Patients (*n* = 20)^a^ (mean ± SD)	Controls (*n* = 20)^b^ (mean ± SD)	*t*	*p*
NAA	9.85 ± 1.15	9.86 ± 0.76	*t*(38) = −0.005	.996
Glu	8.52 ± 0.71	7.43 ± 1.05	*t*(38) = 3.839	.001^c^
Gln	2.46 ± 0.64	2.43 ± 0.41	*t*(35) = 0.174	.863
tCr	8.19 ± 0.74	8.38 ± 0.83	*t*(38) = −0.768	.447
tCho	2.24 ± 0.55	2.43 ± 0.53	*t*(38) = −1.118	.270

NAA, *N*‐acetylaspartate; Glu, glutamate; Gln, glutamine; tCr, total creatine; tCho, total choline.

a NAA *n* = 20, Glu *n* = 20, Gln *n* = 18, tCr *n* = 20, and tCho *n* = 20.

b NAA *n* = 20, Glu *n* = 20, Gln *n* = 19, tCr *n* = 20, and tCho *n* = 20.

c *p* < .05.

### 3.3 Relationship between glutamate and clinical characteristics

**Figure 3:**
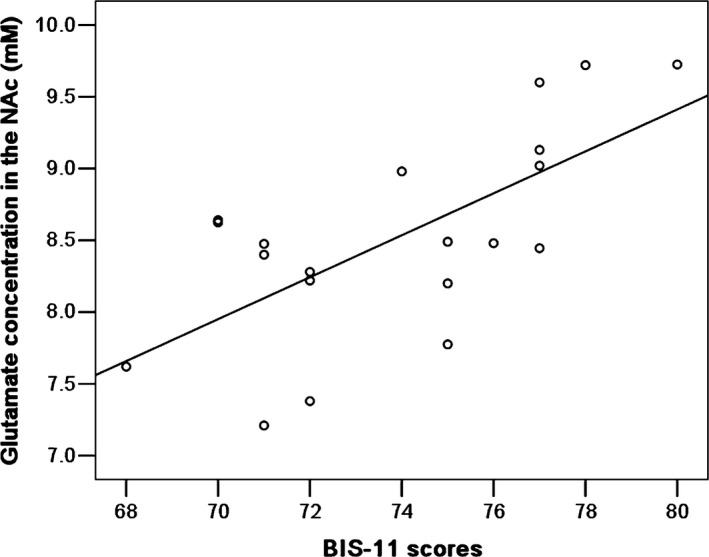
The relationship between the Barratt Impulsiveness Scale (BIS‐11) scores and absolute concentration of glutamate in the nucleus accumbens (NAc) in prescription opiate‐dependent patients

The group comparisons revealed significantly higher glutamate concentrations in the patients. We found a significant positive correlation between absolute concentrations of glutamate and BIS‐11 scores (*r *= .671, *p = *.001, Figure 3). There were no significant correlations between glutamate levels and ASI scores (*r *=* *.079, *p *=* *.742) or SAS scores (*r* = .228, *p *=* *.333). No significant correlations were found between the other metabolite levels and clinical characteristics.

## 4 DISCUSSION

To the best of our knowledge, this study is the first to investigate the in vivo absolute concentration of glutamate in the NAc in prescription opiate‐dependent patients. Although opioids usually are prescribed to control pain, diminish cough, or relieve diarrhea, they also produce feelings of euphoria, tranquility, and sedation that may lead the patient to continue to take these drugs (Schuckit, 2016).

The NAc is a key region that is implicated in the brain's reward circuit, part of a system of structures mediating the reinforcing effects of opiates (Olds, 1982). The NAc is located in the basal forebrain and consists of two primary segments: a medial “shell” subregion and a more lateral “core” component (Mavridis et al., 2011). The NAc serves as a limbic–motor interface (Floresco, 2015). The shell is more related to the limbic system and the core to the extrapyramidal motor system (Neto, Oliveira, Correia, & Ferreira, 2008). Medium spiny neurons, which constitute >90% of the NAc neurons, are characterized by the combined innervations by glutamatergic afferents from the amygdala, frontal cortex, and hippocampus, and dopaminergic afferents from the ventral tegmental area (VTA) (Russo & Nestler, 2013). Glutamate can be coreleased with dopamine in the NAc by VTA dopaminergic neurons expressing vesicular glutamate transporters (VGLUT) (Hnasko, Hjelmstad, Fields, & Edwards, 2012). Together, these inputs provide spatial and contextual information, determine degree of attention allocated to stimuli, inhibit impulsive behavior, and regulate motivational and emotional responses to stimuli (D'Souza, 2015). A multitude of studies in animals and humans implicated the NAc in directing attention and behavior toward appetitive stimuli, including natural rewards such as food and sex as well as opiates. Thus, a pivotal role for the NAc is established for the acute reinforcing effects of addiction.

Long‐term repeated administration of opioids can cause long‐lasting structural and functional changes in neurons. The glutamate system of the brain is responsible for the long‐term plasticity associated with learning and memory. Therefore, it is not surprising that the same glutamatergic mechanism also is implicated in addiction‐related behavior (Camí & Farré, 2003).

Glutamate is the most abundant excitatory neurotransmitter in the mammalian central nervous system and accounts for approximately 70% of synaptic transmission in the brain. It is the principal excitatory neuronal signaling transmitter for many important normal brain functions, including memory, learning, and cognition (Platt, 2007). Glutamate homeostasis in the brain and its deregulation are related to normal and abnormal behavioral adaptations to the environment, respectively (Quintero, 2013). Integration of glutamatergic and dopaminergic neurotransmission is thought to underlie reward‐related learning in corticostriatal networks. There is a substantial amount of literature suggesting that opiates interact with glutamatergic transmission (Gass & Olive, 2008). Most in vitro and in vivo studies have shown that morphine suppresses both basal and evoked increases in extracellular glutamate in NAc and other regions (Sepulveda, Hernandez, Rada, Tucci, & Contreras, 1998). Morphine can also act postsynaptically to suppress glutamate‐evoked neuronal excitation (Giacchino & Henriksen, 1998). In our study, we found significantly higher glutamate levels in the NAc in the prescription opiate‐dependent patients compared to the controls.

Animal research on this subject is mixed. In some published work, administration of addictive drugs like heroin, cocaine, nicotine, or alcohol in both drug‐naïve and drug‐experienced animals, there is an increase in the levels of glutamate in NAc (D'Souza, 2015). Using in vivo microdialysis, glutamate levels have been reported to increase in the NAc in drug‐naïve animals after injection of cocaine (Reid, Hsu, & Berger, 1997), nicotine (Lallemand, Ward, Dravolina, & De Witte, 2006; Liu et al., 2006; Reid, Fox, Ho, & Berger, 2000) and alcohol (Dahchour, Hoffman, Deitrich, & de Witte, 2000). Presentation of cues predictive of cocaine availability increased glutamate levels in NAc in cocaine‐experienced animals. Importantly, heroin‐associated cues have also been shown to increase glutamate levels in the NAc core (LaLumiere & Kalivas, 2008). However, other research suggests that administration of heroin does not increase NAc glutamate levels in drug‐naïve rats. And no change in glutamate levels was observed after cocaine and alcohol injection in drug‐naïve animals, at doses that produce rewarding effects (Dahchour, Quertemont, & De Witte, 1994; Miguens et al., 2008). An increase in glutamate levels was observed downstream from the NAc in the ventral pallidum during heroin self‐administration rats (Caille & Parsons, 2004). Overall, effects of opiate on NAc glutamate levels are not clear.

Proton magnetic resonance spectroscopy is a noninvasive neuroimaging technique that allows in vivo quantification of metabolites. It provides information on the neurophysiologic integrity of brain tissue. Few spectroscopy studies have focused on glutamatergic metabolism in opiate‐dependent individuals. Most of these studies have investigated the anterior cingulate cortex (ACC), and show mixed results. Hermann et al. (2012) found higher glutamate + glutamine (Glx) levels in ACC in older opiate users, and Greenwald et al. (2015) also reported glutamate levels in the ACC were higher at the low relative to the high methadone dose in heroin‐dependent subjects. Murray et al. (2016) reported no significant differences in the glutamate levels in the ACC and a significant decrease in the glutamate levels in dorsolateral prefrontal cortex. Yucel et al. (2007) found the opiate‐using group to have reduced concentrations of dorsal ACC *N*‐acetylaspartate and Glx.

The glutamate signal in MRS consists of complex multiplets due to the scalar couplings and is spread over a wide chemical shift range and superimposed by other signals. These superimposed signals are barely detectable at 1.5T and are still not easily quantified and separated at 3T. The sophistication and utility of MRS studies has been improving in recent years, and confident results are more reliably achieved with optimized sequences (Wijtenburg & Knight‐Scott, 2011). Optimal echo time methods easy acquisition and processing, and appropriate parameter timings can be selected at the scanner interface (Ramadan, Lin, & Stanwell, 2013). Studies using 3.0T scanners with optimized acquisition parameters have produced increasingly reliable and distinct glutamate signals (Hancu, 2009; Mullins et al., 2008; Wijtenburg & Knight‐Scott, 2011).

Our study provides evidence that in the NAc glutamate levels correlate to self‐report impulsivity. The glutamate levels in the NAc were positively correlated with the BIS‐11 scores, which report the behavioral aspect of opiate craving. Impulsivity is common in drug‐dependent individuals and is commonly associated with opiate dependence. Those with higher BIS scores had significantly higher levels of craving demonstrating that impulsivity may impact subsequent drug taking (Mahoney et al., 2015). The relationship between impulsivity and substance abuse is synergistic. Hence, substance abuse seems to be more prevalent among populations that score higher in impulsivity. In this regard, opiate abusers with higher impulsive behaviors are more likely to relapse (Paydary et al., 2016).

In neuropharmacology studies, findings have implicated glutamate neurotransmission in impulsivity. In the 5‐choice serial reaction time task (5CSRTT), systemic injections of nonselective *N*‐methyl‐d‐aspartate (NMDA) receptor antagonists such as dizocilpine (MK801) and ketamine increase impulsive action (Mirjana, Baviera, Invernizzi, & Balducci, 2004). In addition, a novel selective NMDA 2B receptor subunit (NR2B) antagonist Ro 63‐1908 also increased impulsivity as assessed by the 5CSRTT (Higgins, Ballard, Huwyler, Kemp, & Gill, 2003). In addition to NMDA receptors, metabotropic glutamate receptors have been shown to modulate impulsivity (Pattij & Vanderschuren, 2008).

In terms of neuroanatomical localization, alteration of glutamate transmission in the medial prefrontal cortex and its infralimbic region has been associated with impulsive action. Based on MRS studies, glutamate concentrations in the ACC correlated with impulsivity in borderline personality disorder (Hoerst et al., 2010) and attention‐deficit hyperactivity disorder (Ende et al., 2016). In addition, Bauer et al. (2013) reported increasing glutamate levels in the NAc in alcohol‐dependent patients, with glutamate levels in the NAc and the ACC both strongly correlated with the level of the Obsessive Compulsive Drinking Scale. In our study in opiate‐addicted subjects, however, we found that glutamate levels were not significantly correlated with the SAS scores and ASI scores.

Several studies have demonstrated that glutamate is increased in the ACC in patients with general anxiety disorder (Strawn et al., 2013) or social anxiety disorder (Pollack, Jensen, Simon, Kaufman, & Renshaw, 2008). Excess glutamate in anxiety is not only found in categorical comparisons, but also correlates with anxiety severity. This correlation might depend on the type of opioid abused. In our study, all patients were addicted to codeine‐containing cough syrups. Codeine dependence is different from other illicit opioid drugs. The withdrawal symptoms from codeine are lighter than those of other opioids such as morphine and heroin. Codeine‐containing cough syrups contain a combination of codeine, a sympathomimetic, and an antihistamine, all of which have central nervous system action (Qiu et al., 2015, 2016).

There are several important limitations to this investigation. First, in this study, all but one of the patients—and the large majority of controls—were smokers. We found no significant differences in the number of cigarettes between patient and control groups. Hence, the differences in glutamate levels between the groups are unlikely to be explained by smoking. Nevertheless, some animal studies have shown that the administration of nicotine increases glutamate levels in various brain regions (Gass & Olive, 2008). Microdialysis studies suggest that nicotine increases glutamate release in both the VTA and NAc (D'Souza & Markou, 2013; Fu, Matta, Gao, Brower, & Sharp, 2000; Reid et al., 2000). Upregulation of ionotropic glutamate subunits in the prefrontal cortex and the VTA, but not in the NAc, has been reported in chronically nicotine self‐administering rats. In this study, we cannot completely exclude the effects of nicotine. Second, the small sample size may have restricted our power to detect differences, and we used a single voxel to detect glutamate levels of the NAc. Thus, neurochemical differences may have been present in other regions that were not examined in this study, such as other components of the rewards circuit (e.g., VTA, frontal cortex, and amygdala). Finally, we optimized our MRS to detect glutamate in a relatively small VOI with low signal‐to‐noise ratio. Methodological limitations might be responsible for some of the differences in metabolite levels compared with previous studies. And we did not separate gray matter from VOI. Previous studies have reported that there are no change in volume of the NAc structure in patients with prescription opioid dependence (Upadhyay et al., 2010) or marijuana use (Weiland, Thayer, & Depue, 2015). But, Seifert et al. (2015) reported that there might be structural differences in the NAc of heroin‐dependent patients in comparison with healthy controls. Therefore, future studies should examine these data with more exclusive measurements of the NAc gray matter.

## 5 CONCLUSION

Our results show that glutamate levels are elevated in the NAc in prescription opioid‐dependent patients, and that there is a positive correlation between glutamate concentrations and the patient's impulsive behavior. The patient's self‐reported impulsivity relates to the predictability index of craving and relapse. This result suggests that the excitatory neurotransmitter glutamate may play an important role in the neurobiological mechanisms of opiate dependent. With additional work, the absolute glutamate concentrations in the NAc measured quantitatively with in vivo ^1^H MRS could become a useful biomarker to assess the likelihood of relapse. The spectroscopic methodology proved to be a reliable and useful approach for investigating the glutamate in vivo in the brain.

## CONFLICT OF INTEREST

All authors have no conflict of interest to report.
